# Study on the process of mass transfer and deterioration of limestone under dynamic dissolution of CO_2_ solution

**DOI:** 10.1038/s41598-024-56038-w

**Published:** 2024-03-04

**Authors:** Wushu Dong, Ze Li, Linfang Shen, Wenlian Liu, Yakun Guo, Hanhua Xu, Rui Yong

**Affiliations:** 1 Faculty of Civil Engineering and Mechanics, Kunming University of Science and Technology, Kunming, 650500 Yunnan China; 2 Kunming Prospecting Design Institute of China Nonferrous Metals Industry Co., Ltd, Kunming, 650051 China; 3 Faculty of Engineering and Informatics, University of Bradford, Bradford, BD7 1DP UK; 4 Yunnan Key Laboratory of Geotechnical Engineering and Geohazards, Kunming, 650051 China; 5 School of Civil and Environmental Engineering, Ningbo University, Ningbo, 315211 Zhejiang China

**Keywords:** Limestone, Dynamic corrosion, Macroscopic mechanical property deterioration, Mass transfer—deterioration mechanism, Civil engineering, Natural hazards

## Abstract

The long-term erosion of rock by solution can induce a series of karst problems. Therefore, this study focused on limestone and conducted dynamic dissolution experiments under deionized water and CO_2_ solution conditions to study the deterioration mechanism of limestone under nonequilibrium conditions. The results showed that the degree of degradation of the mechanical properties of the samples in a CO_2_ solution was obviously greater. In a deionized water environment, the degradation of the mechanical properties of the sample is mainly controlled by the physical softening action of the solution. In the CO_2_ solution environment, the degradation process can be divided into two stages. In the early stage of the experiment (10 days to 20 days), the degradation of mechanical properties of the sample is also controlled by the physical softening action of the solution. With increasing soaking time, the main rock-forming minerals of limestone gradually react with the CO_2_ solution, the degradation of the sample is controlled mainly by the chemical corrosion of the CO_2_ solution, and its degradation rate is much greater than that of physical softening. The results can be used as a reference for assessing the long-term stability of underground engineering in limestone karst development areas.

## Introduction

Soluble rocks (mainly limestone) are widely distributed in China, with a distribution area of up to 3.65 million km^2^, accounting for approximately one-third of the country’s land area. In particular, the total area of underground soluble rocks in the southwestern region is approximately 41.05 × 104 km^2^, accounting for 30% of the total rock area. The long-term water and gas circulation inevitably cause a series of dissolution effects, which generate karst landforms such as underground caves, sinkholes, and dissolution fractures, ultimately affecting the macroscopic physical and mechanical properties of rock layers. Therefore, when karst development areas are used as foundation bearing layers, the degree of underground rock dissolution development directly affects the safe and effective operation of upper building facilities^1^.

**Figure 1:**
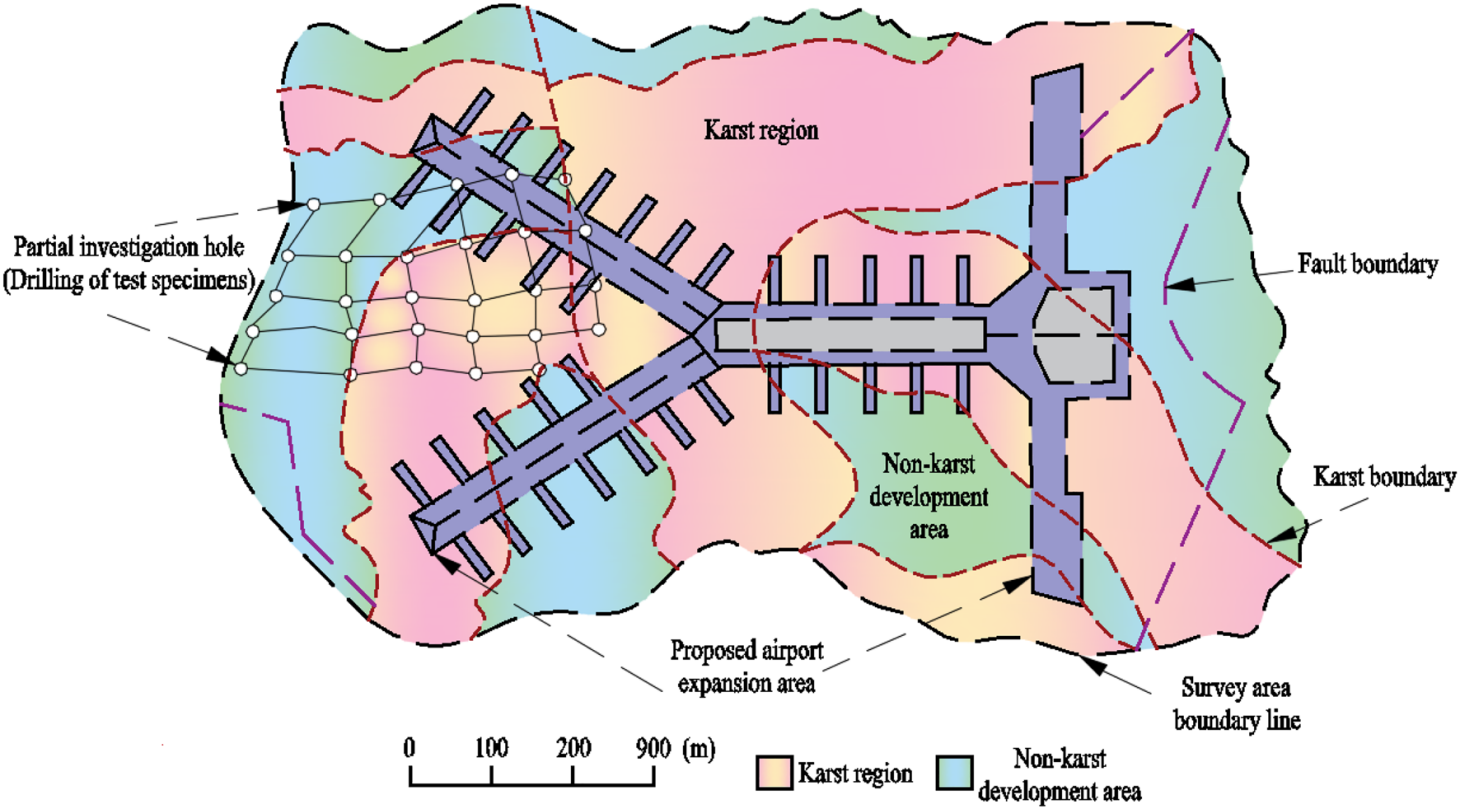
The geological characteristics of the planned expansion area.

The T2 terminal expansion project of Kunming Changshui International Airport in Yunnan Province is a key planning project of the Civil Aviation Administration of China (CAAC), and its expansion project is located in the Yunnan Eastern Karst-Qiufeng Plateau subregion. The engineering construction scope was positioned using RTK measurement technology, and 495 boreholes were arranged in the proposed expansion area. Through the drilling process and core property detection, the geological characteristics of this area were surveyed, the survey results are shown in Fig. 1. The fig shows that there is strong karst development in the proposed expansion area. Near the proposed expansion project area, the original Guikun Railway, in the section from Hengshuitang to Yangtianchong, experienced 79 karst collapses from 1975 to 1991 along a 10km stretch, significantly affecting the safe operation of trains.

The design and operation period of the Kunming Changshui International Airport expansion project will last for hundreds of years. The development of underground karst poses a great threat to the stability and durability of overall projects, especially flight areas and terminal building foundations. Therefore, it is necessary to aim at the geological features of the region to conduct simulated experiments on limestone dissolution to analyze its dissolution degradation characteristics.

In response to the issue of limestone dissolution, numerous scholars have undertaken a series of related studies, and their research results can be summarized in several aspects. (1) Influence of moisture content: The moisture content is negatively correlated with the mechanical properties of rocks, i.e., the higher the moisture content of the rock mass is, the lower its mechanical performance^2–8^. (2) Influence of solution properties: The erosion degradation of limestone is mainly controlled by the chemical dissolution of the solution and the mass transfer process of major mineral ions, and the degree of erosion degradation of the rock mass increases with decreasing solution pH^9–22^. (3) Influence of wet-dry cycle effects: During the wet-dry cycle process, the degree of degradation in the macroscopic physical and mechanical properties of limestone increases with the number of cycles^23–25^. (4) Influence of rock properties: Based on the quantitative analysis of the main rock-forming minerals, the erosion reaction rate and mechanical energy degradation rate of different limestone properties are determined under the same solution environment^26,27^.

According to the research results, the macroscopic performance and microscopic structure of limestone deteriorate to varying degrees after being eroded by the solution. However, most of the existing experimental studies on water–rock dissolution processes are established under a single stationary closed equilibrium system, without considering the state of the soaking solution and the saturation and migration of ion concentration. In actual water–rock interaction processes, the solution is mostly in a flowing state. After the mineral ions in the rock matrix enter the solution, they will migrate with the water solution and will not appear in a concentrated state on the rock surface, so the dissolution process will always remain in a nonequilibrium reaction stage. In addition, most of the existing experimental studies use chemically prepared soaking solutions, and there are currently no studies on the damage mechanics of limestone under dynamic dissolution in a CO_2_ solution. However, the dynamic CO_2_ solution environment is the most important dissolution medium in the natural environment^28,29^. The changes in the macroscopic physical and mechanical parameters of limestone obtained under this dissolution environment can effectively reflect the deterioration law of rock mass performance under natural conditions, which has important practical engineering significance.

Research on the deterioration mechanism of limestone has focused mainly on the chemical dissolution of rock-forming minerals in the matrix by chemical solutions. However, in reality, water solutions are not high-concentration chemical solutions, and when they come into contact with rocks, they do not undergo violent chemical reactions immediately. There is an obvious “time effect”, during which the deterioration of rock properties may be controlled mainly by the physical dissolution of water solutions. However, few studies have quantify the deterioration mechanism of limestone from the perspective of physical and chemical dissolution.

In this regard, this paper based on the Kunming Changshui International Airport expansion project survey, independently developed and designed a dynamic dissolution system for CO_2_ solution. A dynamic dissolution simulation experiment for limestone was constructed to investigate and analyze the variation patterns of the uniaxial compressive strength and elastic modulus of limestone specimens after dynamic dissolution for 0 days, 10 days, 20 days, 30 days, 40 days, 50 days, and 60 days. Quantitative analysis of the destruction characteristics of the specimens was conducted based on the relative changes in energy. By utilizing SEM images as a foundation and employing digital image processing techniques, the study statistically analyzed the variation patterns of microscopic damage areas with immersion periods. Finally, based on the changes in Ca^2+^ concentration, this paper elucidates the correlation mechanism of limestone dissolution mass transfer processes under different solution environments and the primary controlling factors of macroscopic degradation of mechanical properties. This research provides experimental references and theoretical support for predicting the service life of underground foundations in expansion projects and designing for weathering resistance.

## Research background

**Figure 2:**
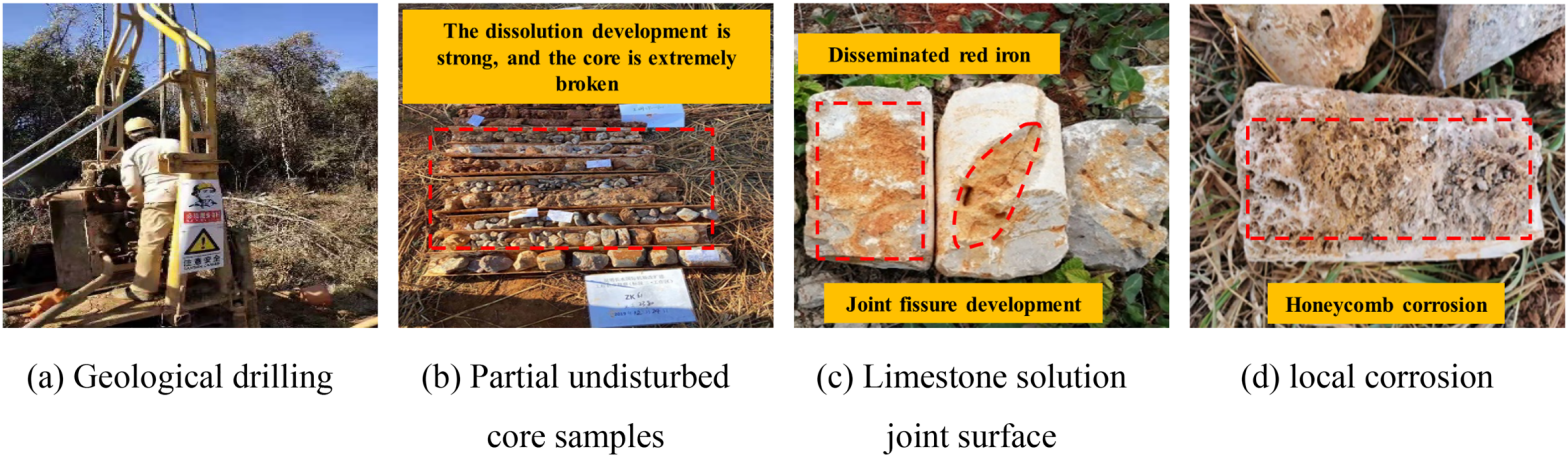
On-site limestone core sample.

This experiment is based on the detailed survey project of the Kunming Changshui International Airport T2 terminal expansion, a key engineering project in Yunnan Province. In 2022, Kunming Changshui International Airport completed transportation takeoffs and landings for 192,700 flights, with a passenger throughput of 21.2375 million and a cargo throughput of 310,000 tons. All these indicators have far exceeded the design capacity, so the expansion of the airport imminent. The engineering expansion area is located in Dabanqiao Town, Guandu District, Kunming City, with an altitude ranging from 2020 to 2347 m above sea level, and a total surveyed area of approximately 15.3 km^2^. Figure 2 shows the core samples extracted on-site, it can be seen that the rock mass is brownish-gray in color, moderately weathered, and has a cryptocrystalline structure. Localized honeycomb-like dissolution pores can be observed in the core, with some rock structures appearing fragmented. The development of joint fissures is significant, and some joint surfaces show infiltration of red iron minerals. Geological tests indicate that the underlying bedrock in this area mainly consists of Permian Yangxin Formation limestone.

**Figure 3:**
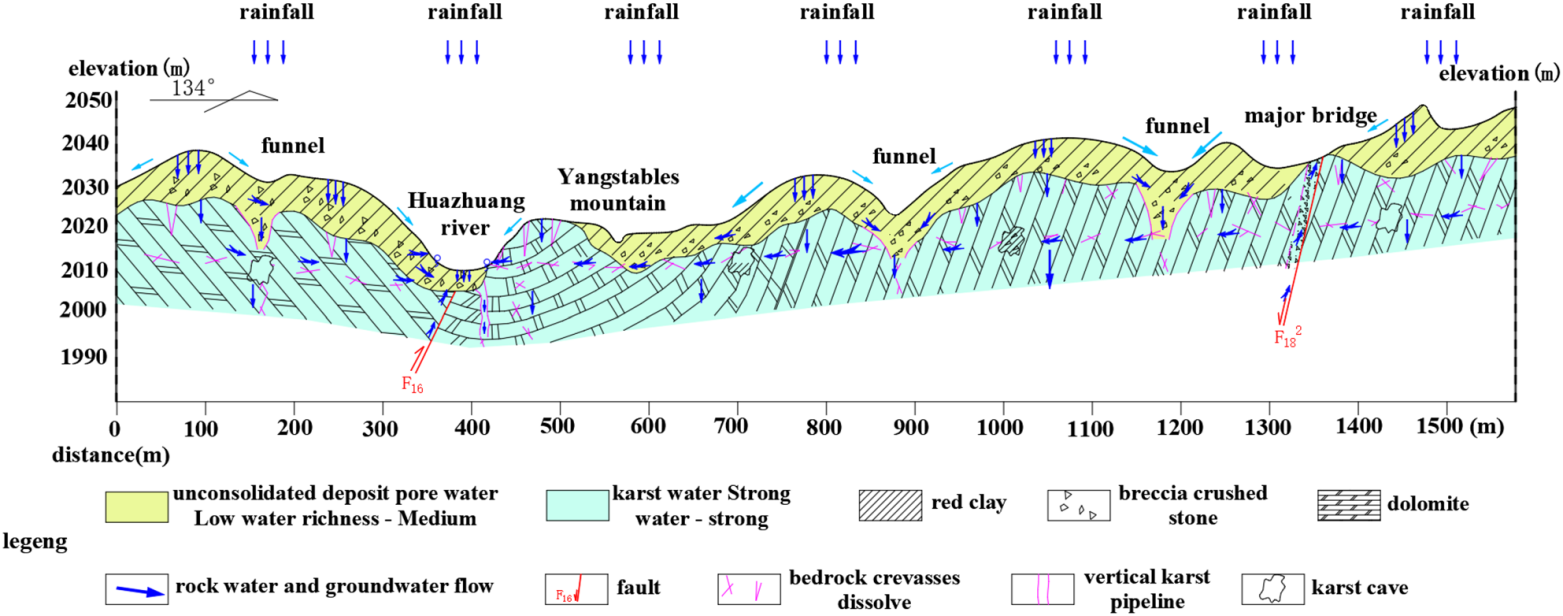
Hydrogeological profile.

**Figure 4:**
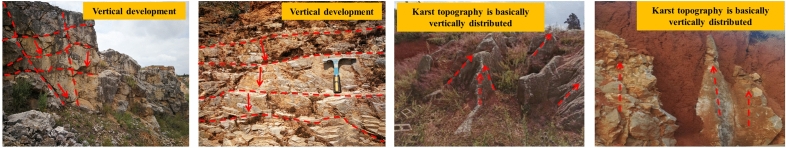
Karst development characteristics.

The development of underground karst is not only determined by the properties of the rock mass, but also controlled by hydrogeological conditions^30–33^. Figure 3 shows the hydrogeological profile of the proposed expansion area of the airport. The fig shows that the rock layer solution in the proposed expansion area mainly originates from the direct infiltration of atmospheric precipitation. The vertical infiltration method further enhances the cyclic interaction between the water solution and the atmosphere, allowing more CO_2_ gas to enter the infiltrating water solution to generate a corrosive CO_2_ solution. According to hydrogeological surveys, the pH of the rock layer water solution and groundwater in this area is maintained between 4.10 and 4.89 throughout the year, showing weak acidity. The main ion elements in the water are HCO_3_^-^, Ca^2+^, and Mg^2+^. Figure 4 shows the geological strata and the morphology of rock dissolution photographed during the geological survey process. The fig shows that karstification primarily develops in the vertical direction. Combining with hydrogeological characteristics, it indicates that the development of underground karst in the planned expansion area is controlled by the combined action of water solution and CO_2_ gas.

## Test materials and methods

### Test material

**Figure 5:**
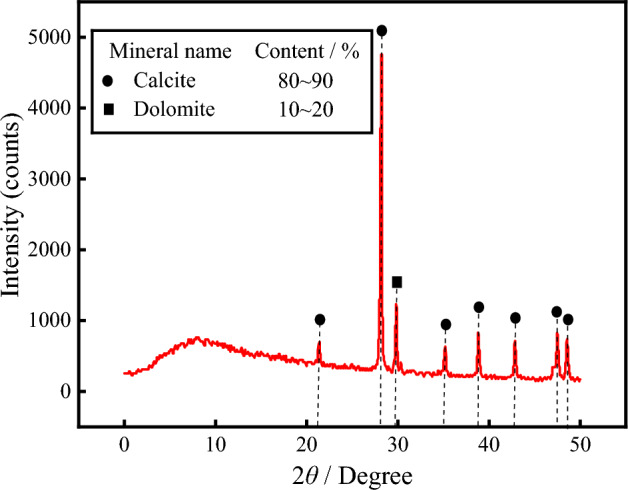
X-ray diffraction analysis of the sample.

The gray rock samples collected from the drilling holes in the proposed expansion area were analyzed via X-ray diffraction (XRD). The mineral matrix of the gray rock mainly consists of calcite and dolomite, with calcite accounting for 80% to 90% and dolomite accounting for 10% to 20%, as shown in Fig. 5. Additionally, X-ray fluorescence spectroscopy (XRF) was performed on the powder samples, and the main elemental oxides were obtained as shown in Table 1. According to the table, the CaO and MgO contents are 82.749% and 0.499%, respectively, which match the results of the main mineral component content test. The Ca element corresponds to calcite and dolomite, while the Mg element corresponds to dolomite, indicating that the gray rock in this area is a typical carbonate rock.

**Table 1:** Mass percentages of the main element oxides in the sampled limestone.

Oxide name	CaO	MgO	SiO_2_	Al_2_O_3_	Fe_2_O_3_
Content/%	82.749	0.499	0.098	0.045	0.029

**Figure 6:**
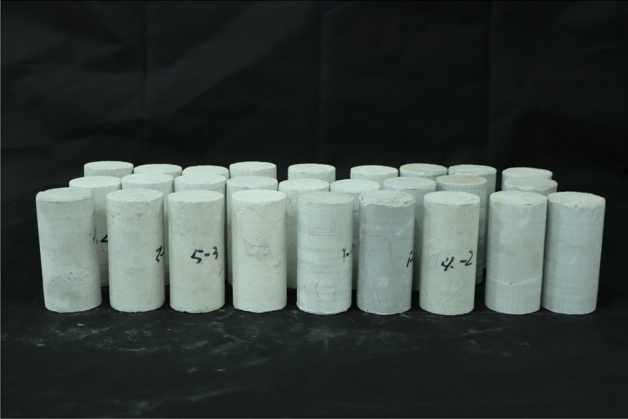
Part of rock samples.

**Figure 7:**
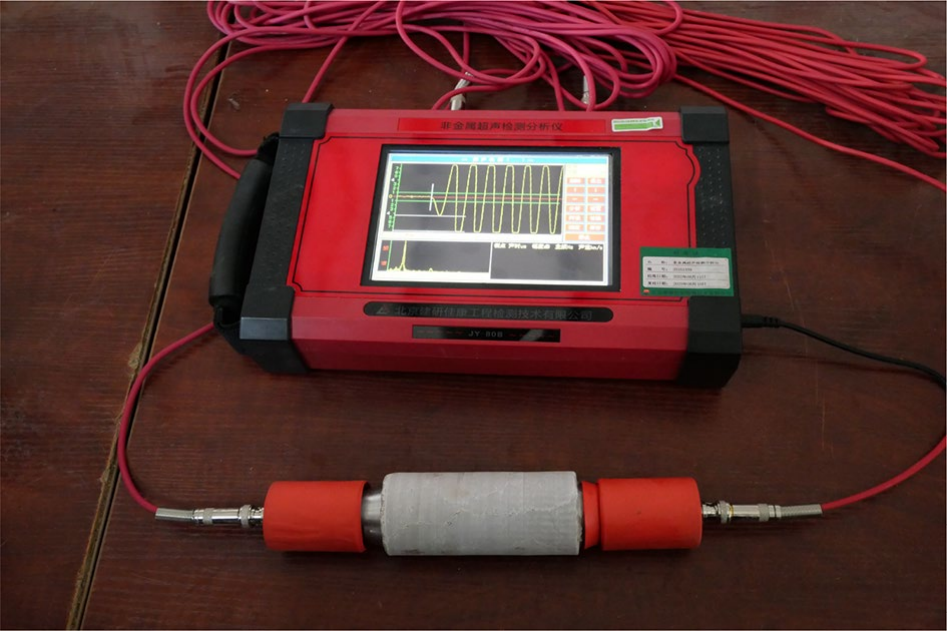
Wave speed screening.

During the process of collecting and selecting samples on site, the original rock samples that had no obvious penetrating cracks or gaps that seriously affected the integrity of the rock mass on the surface were selected. During the transportation process, collision between rock samples should be reduced, and fragile areas such as the edges and corners of the samples should be ensured to maintain their original state without damage. The collected original samples are processed into cylindrical samples with a size of 50 mm × 100 mm, as shown in Fig. 6. To reduce the influence of sample discreteness on the test results, the appearance, specific gravity, and longitudinal wave velocity (as shown in Fig. 7) of the processed samples were screened, and samples with large data differences were eliminated.

### Test methods

#### Dynamic solution system

**Figure 8:**
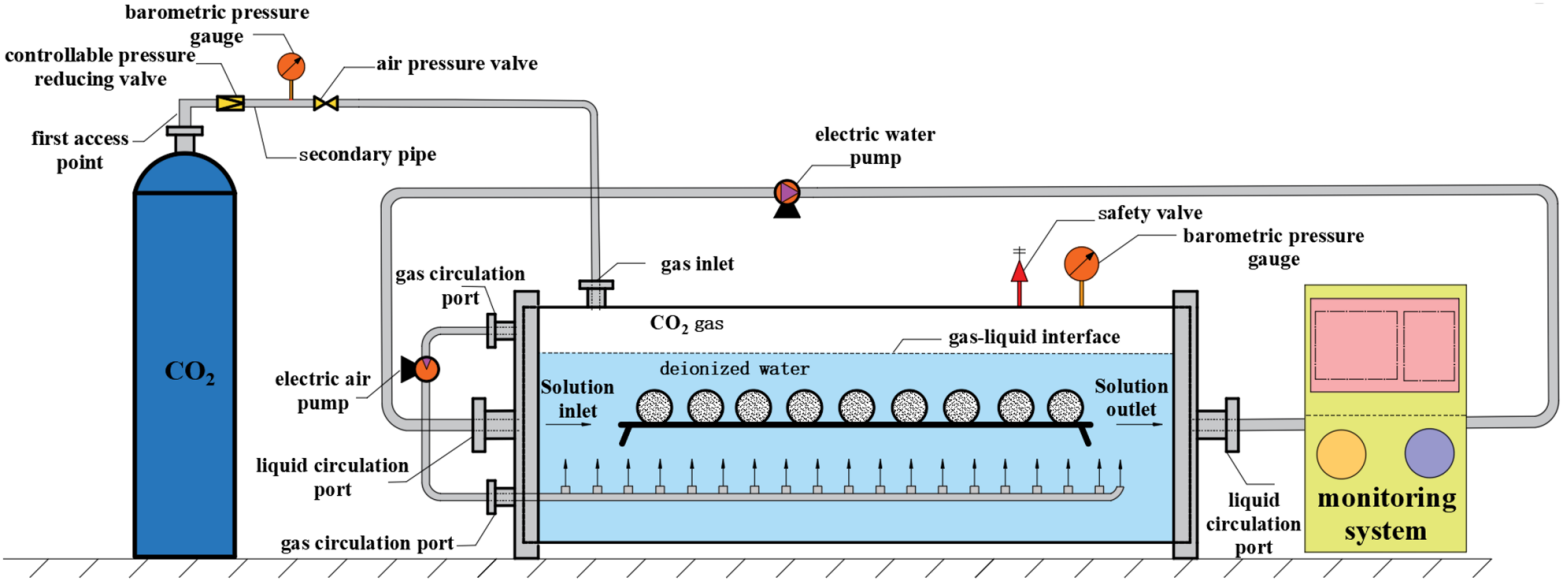
Dynamic dissolution test device.

To effectively analyze the dissolution characteristics of limestone in the proposed construction area, a dynamic dissolution experiment system was independently developed and designed based on the hydrogeological characteristics of this area. As shown in Fig. 8, the system mainly consists of a dissolution reaction box, a CO_2_ circulation system, a solution circulation system, and a solution monitoring system. The dissolution reaction box is water–rock interaction platform, that has multiple channels and couples the CO_2_ circulation system and the solution circulation system through these channels to form a dynamic CO_2_ solution environment inside the dissolution reaction box.

The CO_2_ circulation system consists of a gas input port, a gas circulation port, an electric waterproof air pump, a gas connecting hose, a spring-type air pressure safety valve, and a gas pressure gauge. CO_2_ gas is injected into the dissolution reaction box through the gas input port using a CO_2_ gas cylinder. The circulation suction port, electric waterproof air pump, and circulation exhaust port are connected in sequence through the connecting hose, and the circulation suction port is kept above the gas–liquid interface. Finally, an electric waterproof air pump is driven to ensure that the CO_2_ gas in the reaction box is always in a circulating state, thereby ensuring that more CO_2_ gas is dissolved in the soaking solution to form a corrosive carbonic acid solution.

The solution circulation system includes a solution circulation inlet, an electric water pump, and a solution circulation outlet, which are sequentially connected by solution-conducting hoses. Based on the drive from the electric water pump, the immersion solution is kept in a flowing state, creating a realistic dynamic dissolution environment within the corrosion reaction chamber. Finally, through a monitoring system, real-time detection of changes in the solution flow rate and pH is conducted.

#### Dynamic corrosion system verification

To ensure that the dynamic dissolution system can simulate the water solution environment of the proposed construction area more realistically, a preliminary experiment was conducted before the formal experiment to verify the safety and effectiveness of the dynamic dissolution system.

**Figure 9:**
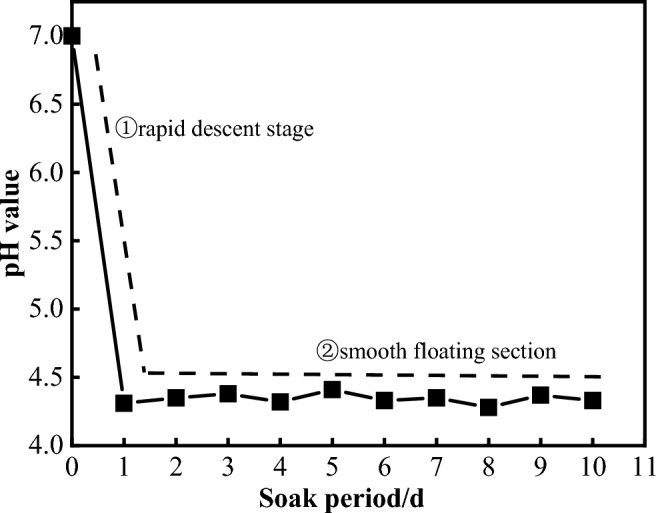
Time history evolution of the solution pH under a partial pressure of CO_2_ 0.1MPa.

The process began by injecting 40 L of deionized water into the dissolution reaction box. Subsequently, carbon dioxide gas was introduced into the dissolution reaction box using a carbon dioxide cylinder. Once the pressure gauge stabilizes at 0.1 MPa, the air pump and water pump are activated to establish the dynamic circulation of deionized water and carbon dioxide gas. Simultaneously, the solution monitoring system was used to perform real-time monitoring of the solution's pH within the reaction box. Figure 9 shows the variation in the solution pH over immersion time. As shown in the fig, the solution's pH underwent approximately two stages of change. After the experimental setup was run for 1 day, the solution's pH rapidly decreased to 4.30. With increasing immersion time, there was a slight fluctuation in the pH, which consistently hovering around 4.30. This indicates that the pH of the solution formed in the dissolution reaction box is basically consistent with that of the underground water in the proposed construction area.

**Figure 10:**
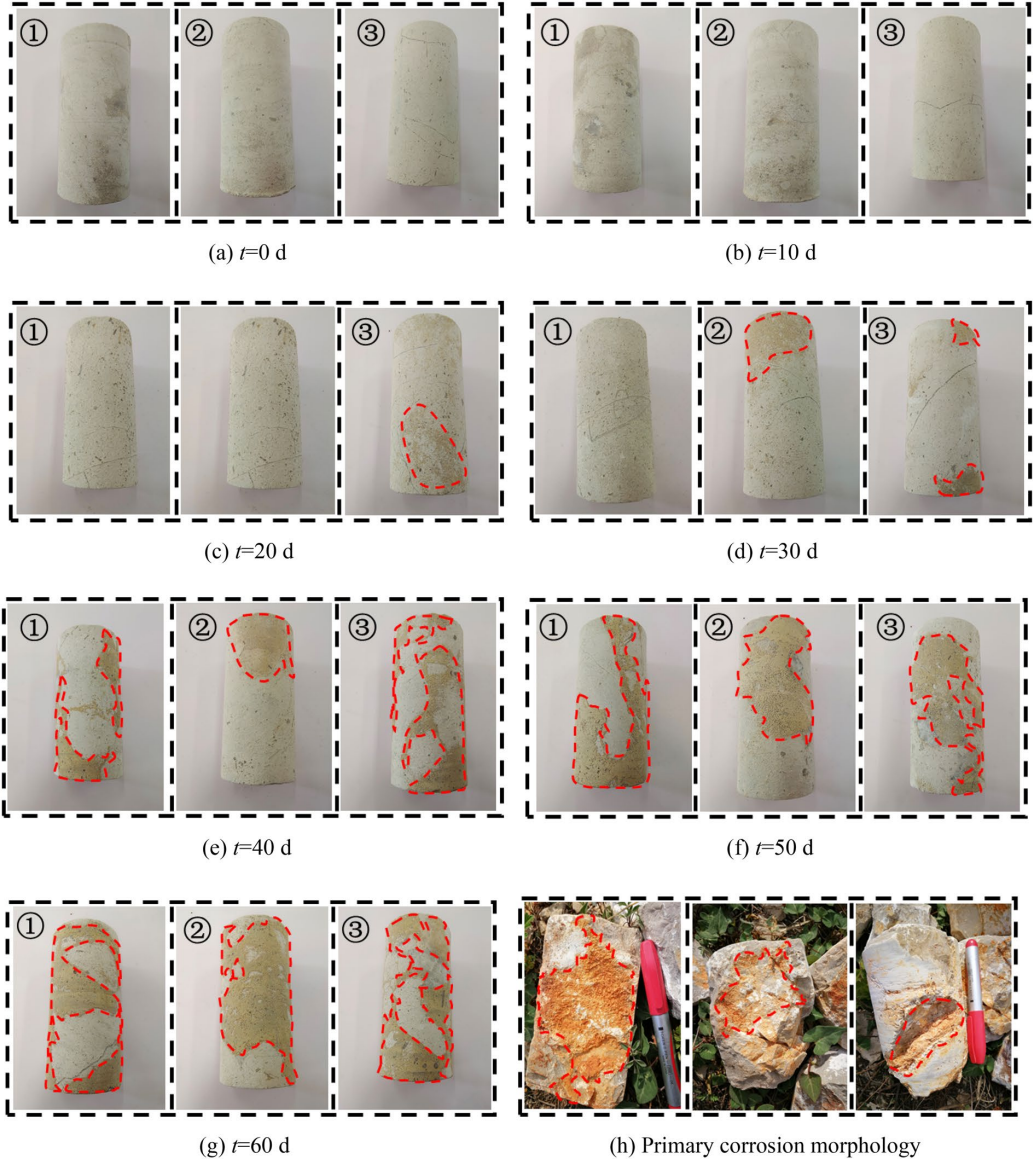
Variation in the apparent morphology of limestone samples over time.

During the process of karst development, the physical and chemical dissolution of water solutions can cause varying degrees of deterioration on the surface of the rock mass. Therefore, to verify that the dissolution effect generated by the dynamic dissolution system is similar to that of the proposed construction area, three gray rock samples were used to conduct dynamic dissolution experiments, and changes in their apparent morphology were observed. Figure 10a–g show the temporal evolution of the apparent morphology of the gray rock samples under the dynamic dissolution of CO_2_ solution. The fig shows that the surface of the original sample without dissolution is relatively smooth and dense. With increasing immersion time, the sample surface gradually becomes “rougher” and deteriorates to varying degrees, accompanied by the shedding of a large amount of dust.

After 20 days of dissolution, the surface of the sample showed a honeycomb-like morphology similar to the primary dissolution morphology of the underground limestone in the proposed construction area (Fig. 10h). With increasing immersion time, this dissolution morphology feature was continuously enhanced and was basically consistent with the primary dissolution morphology of the limestone in the proposed construction area after 60 days. This indicates that the designed dynamic dissolution system can effectively simulate the underground dissolution environment of the proposed construction area and lay the foundation for studying the deterioration characteristics of limestone in this area.

#### Dynamic corrosion experiment

To study and analyze the dissolution characteristics and deterioration mechanism of limestone in the proposed construction area, the following steps were taken to conduct dynamic dissolution experiments on limestone based on the above dynamic dissolution system:

1. Before the dissolution test, the limestone samples were placed in an oven and dried for 24 h at a temperature of 105 ℃. After the sample temperature decreased to room temperature, the mass was recorded.
2. The sample was placed in the dissolution reaction box, and 40 L of deionized water was injected into the dissolution reaction box to ensure that the sample was completely immersed. The electric water pump was turned on to keep the deionized water in a flowing state.
3. The inflation valve was opened to inject CO_2_ gas into the dissolution reaction box until the upper pressure gauge on the reaction box stabilized at 0.1 MPa. During the CO_2_ gas injection process, the gas cylinder pressure was kept between 0.05 and 0.1 MPa. Finally, the electric air pump was turned on to form a circulating CO_2_ gas, which fully dissolved in deionized water to form a saturated CO_2_ solution, thereby achieving dynamic dissolution of the gray rock.

A 10-day is designated as one experimental cycle, with a total of 7 stages (0 days, 10 days, 20 days, 30 days, 40 days, 50 days, and 60 days). To prevent the ion concentration of the immersion solution from reaching saturation during the experiment and impeding the dissolution of the rock, the deionized water solution was replaced every 10 days, and the aforementioned experimental procedures were repeated. Additionally, a control group experiment was set up under the same experimental cycle, where no CO_2_ gas was introduced in the control experiment, and only the flow of deionized water was maintained.

**Figure 11:**
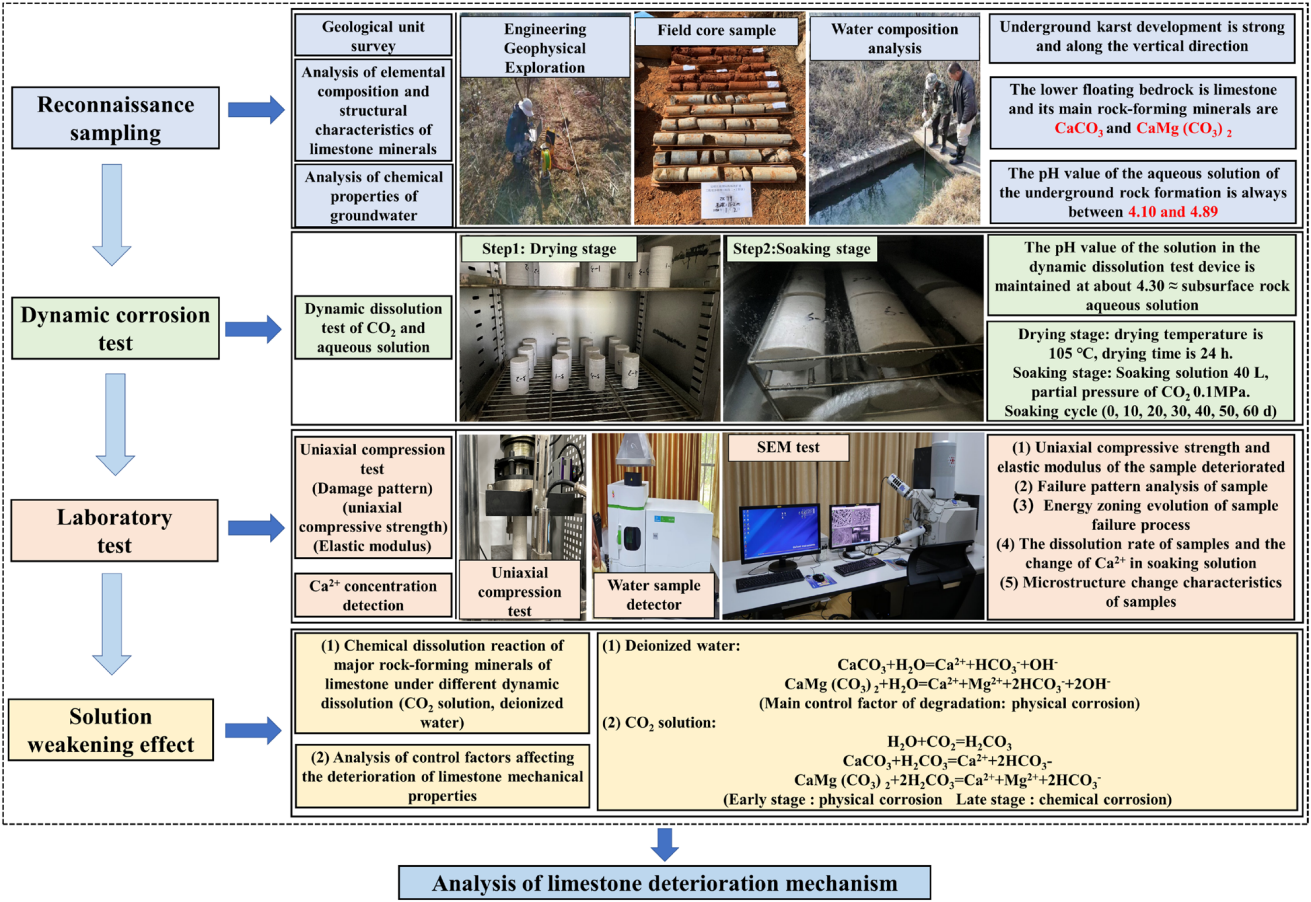
Design diagram of the test flow.

To investigate the deteriorative effects of the dynamic dissolution of CO_2_ and water solutions on limestone specimens, as well as the water–rock interaction mechanisms, various tests were conducted after different immersion periods in deionized water and CO_2_ solutions. The parameters examined included uniaxial compressive strength, elastic modulus, mass loss rate, scanning electron microscopy (SEM) images of the corroded specimen surfaces, and changes in the Ca^2+^ concentration in the immersion solution. The experimental procedure is shown in Fig. 11. The equipment and testing methods involved in the experiments are outlined below:

1. Uniaxial compression test: Uniaxial compression tests were conducted using a YAW-2000D microcomputer-controlled electrohydraulic servo pressure testing machine on 50 mm × 100 mm specimens that had undergone the corresponding immersion period. Three parallel specimens were tested, and the loading rate was set at 0.5 MPa/s.
2. Scanning electron microscopy (SEM) observation: After the uniaxial compression test, fragments of the specimens that were in contact with the immersion solution were selected for observation. According to the SY/T516-2014 "Scanning Electron Microscopy Analysis Method for Rock Samples" standard, a small amount of thin slices was cut, made into observation specimens, and subjected to gold spray treatment. Subsequently, a Nova Nano SEM450 high-resolution field emission scanning electron microscope was used to observe the changes in microscopic structure and morphology.
3. Ca^2+^ concentration testing: The Ca^2+^ concentration in the immersion solution at each experimental stage was measured using inductively coupled plasma emission spectroscopy (HJ776-2015).

## Test results and analysis

### Mechanical properties of limestone

**Figure 12:**
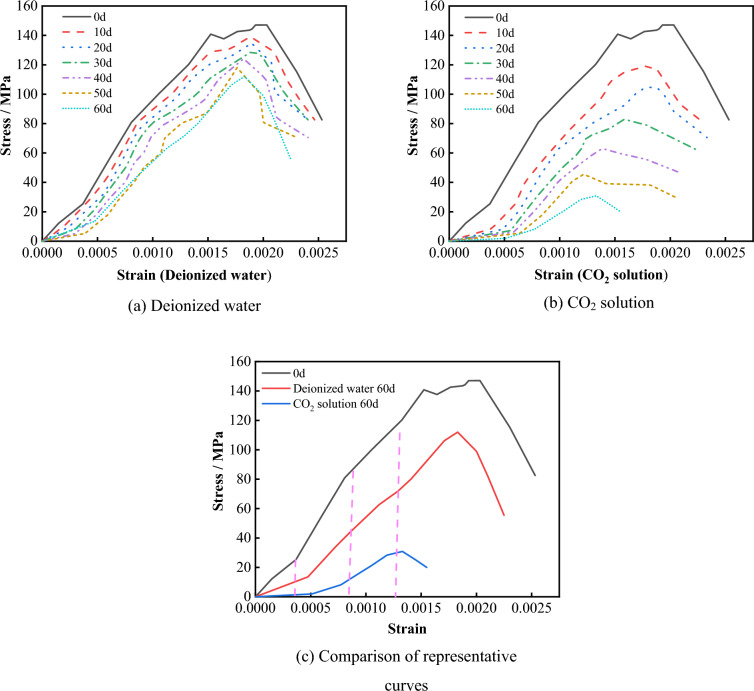
Changes of stress and strain of limestone samples under various solution.

A series of uniaxial compression tests were carried out on limestone samples under different conditions to investigate the variations in stress and strain, as shown in Fig. 12. It can be seen that the deformation characteristics of the limestone stress–strain curves are influenced by both the immersion time and the nature of the immersion solution. For intact specimens without prior testing, the stress rapidly decreases after reaching the peak stress, showing a pronounced brittle behavior. As the immersion time increases, the stress–strain curves of specimens immersed in deionized water and CO_2_ solution both exhibit varying degrees of downward shifting. The rate of post-peak stress decay decreases, the phase change rate of post-peak strain gradually increases, and the plastic characteristics of the specimens gradually enhance. Furthermore, under the same experimental period, taking the example of 60 days of corrosion (Fig. 12c), the changes in the stress–strain curves of specimens in a CO_2_ solution environment are particularly significant, showing a substantial downward shift. This suggests that prolonged immersion leads to the entry of aqueous solution into the rock, creating a certain pore water pressure, thereby promoting the development and extension of cracks. Simultaneously, the chemical dissolution effect of the CO_2_ solution exacerbates the internal structural damage of the rock, making it more susceptible to the rapid development and extension of cracks under loading, ultimately resulting in a significant deterioration in specimen strength.

**Figure 13:**
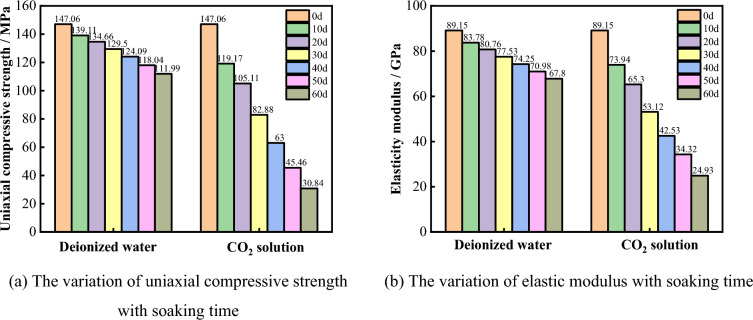
Deterioration process of the mechanical properties of the samples.

To accurately quantify the degradation pattern of limestone mechanical properties under dynamic dissolution in deionized water and CO_2_ solution, a statistical analysis based on stress–strain curves was conducted to analyze the variations in the uniaxial compressive strength and elastic modulus of the specimens under different experimental conditions, as shown in Fig. 13. It can be seen that, compared to the natural state, the uniaxial compressive strength and elastic modulus of the specimens decrease with increasing immersion time. However, the degree of degradation varies, influenced by different environments. After 60 days of immersion in deionized water, the uniaxial compressive strength of the specimen decreased to 111.99 MPa, a reduction of 23.85%, and the elastic modulus decreased to 67.80 GPa, a reduction of 23.95%. After 60 days of immersion in CO_2_ solution, the uniaxial compressive strength decreased to 30.84 MPa, a reduction of 79.03%, and the elastic modulus decreased to 24.93 GPa, a reduction of 72.03%.

The above results indicate that the degradation of limestone mechanical properties by deionized water and CO_2_ solution is positively correlated with immersion time, but the degradation effect of CO_2_ solution is greater than that of deionized water. The reason for this phenomenon is that limestone, as a typical sedimentary rock, is primarily composed of various mineral cements. The carbonic acid solution generated by CO_2_ gas reacts chemically with some soluble minerals, leading to a gradual decrease in the mechanical properties of limestone. In a deionized water environment, limestone minerals undergo only a low degree of hydrolysis and ionization, resulting in a lower rate of degradation of its mechanical properties.

### Damage morphological characteristics of limestone

**Figure 14:**
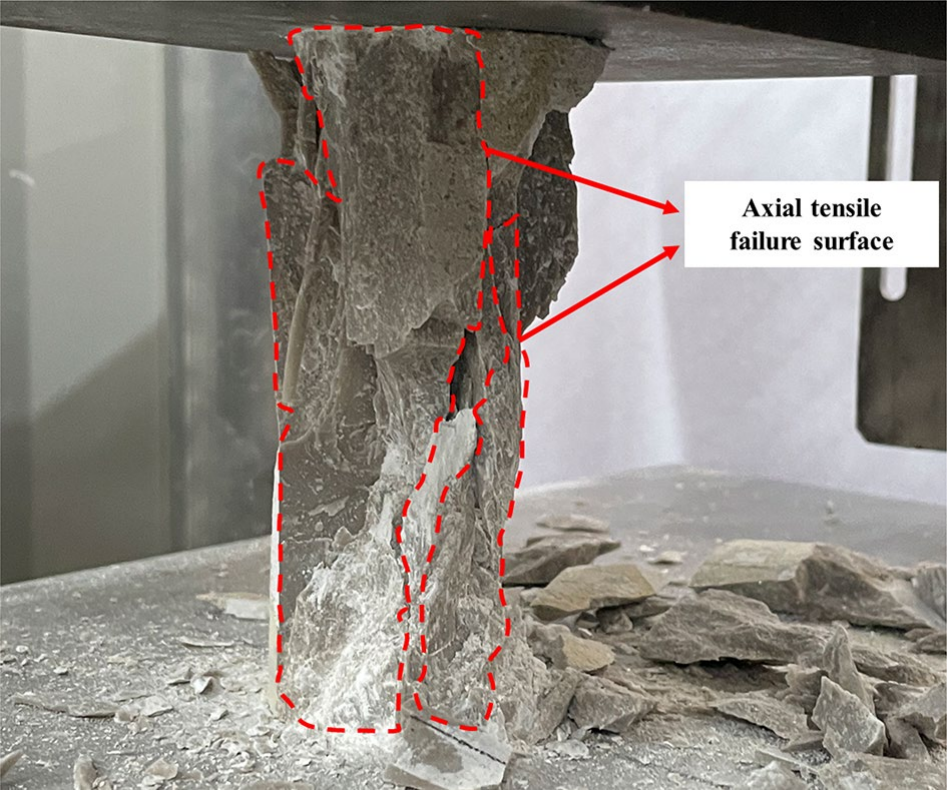
Failure morphology of undisturbed limestone samples under uniaxial compression (*t* = 0 d).

To analyze the failure characteristics of limestone specimens under different experimental conditions, industrial cameras were used to capture the failure morphology of specimens after uniaxial compression tests. Figure 14 shows the failure morphology of the intact specimens without dissolution. It can be observed from the fig that the specimens exhibit severe splitting failure, with the failure surface being the axial tensile failure surface that penetrates the specimen. Additionally, during the failure process, there were accompanying loud explosions, indicating obvious brittle failure properties.

**Figure 15:**
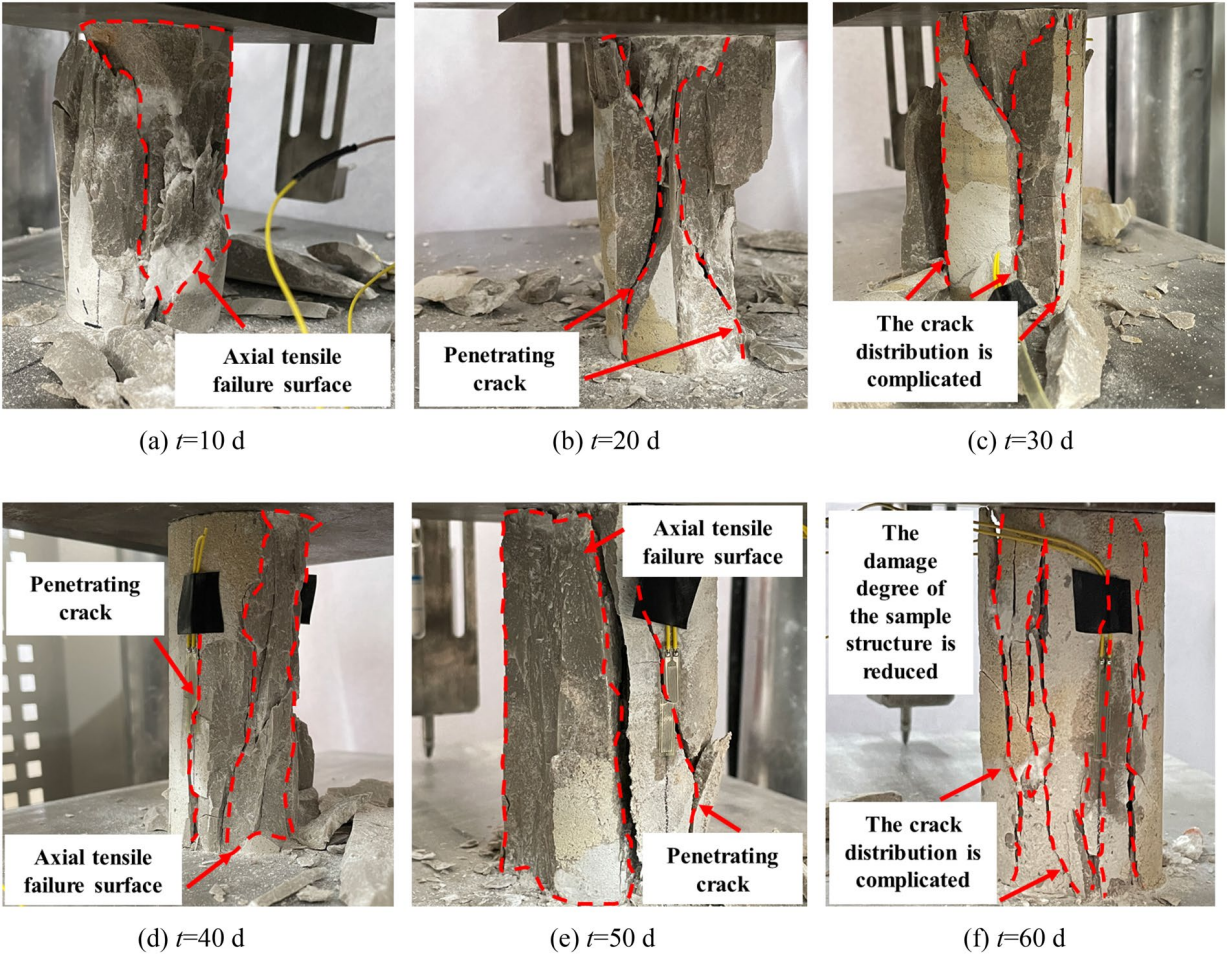
Uniaxial compression failure morphology of limestone after immersion in deionized water.

Figure 15 shows the failure morphology of the specimens after immersion in deionized water. It can be observed from the fig that the failure morphology of the specimens is similar to that of the intact specimens, indicating brittle failure. However, with increasing immersion time, the brittle failure properties decrease, and plastic failure properties gradually appear. The degree of specimen failure decreases, and the failure sound becomes mixed and subdued.

**Figure 16:**
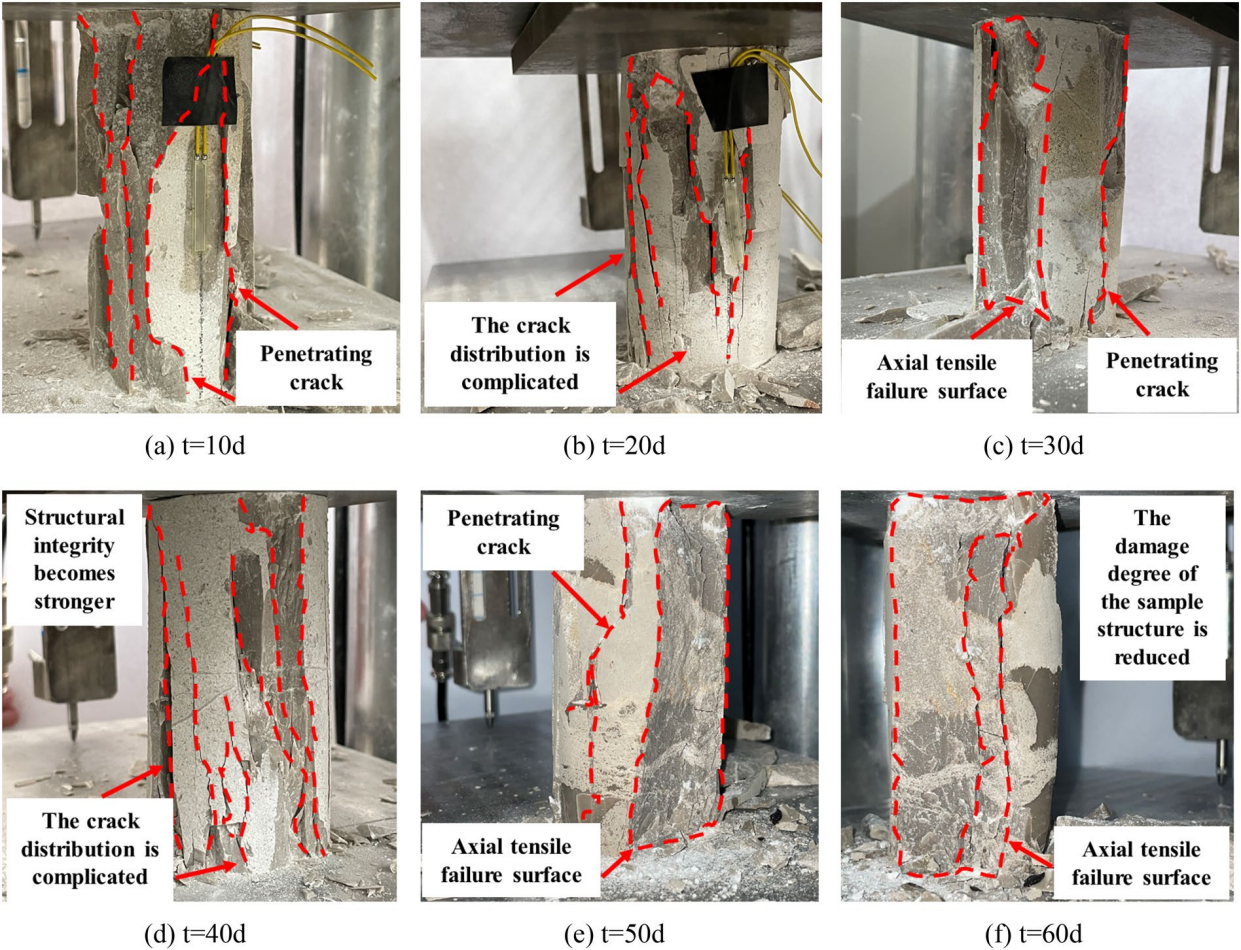
Uniaxial compression failure morphology of limestone after CO_2_ solution immersion.

Figure 16 shows the failure morphology of the specimens after immersion in CO_2_ solution. It can be observed from the fig that the plastic deformation characteristics of the failure mode of the samples are most pronounced. Due to the erosive action of the CO_2_ solution, numerous irregular pores are formed within the specimen, altering its original dense structure. Under axial loading, numerous vertical cracks appear on the main surface of the specimen, accompanied by local shear deformation. Additionally, the CO_2_ solution further develops the pre-existing fracture surfaces, creating more structural defect areas. As a result, the fracture surface of the specimen increases, and the fracture morphology becomes more complex. In some cases, the surface even separates from the main body, ultimately leading to a loss of load-bearing capacity in the specimen.

However, due to the presence of numerous nonlinear discontinuity planes and differences in mineral composition within the specimen, its failure morphology is complex and diverse. Direct observation alone can provide only a general rule of the failure process, with limited persuasiveness. However, during the damage, deformation, and compression failure processes of rocks, energy accumulation and dissipation often occur. Therefore, based on the theory of energy evolution, it is possible to quantify the failure characteristics of rock specimens under different experimental conditions, providing a better understanding of their damage development patterns.

### The law of energy partition evolution of samples

During the uniaxial compression process, the deformation of rock specimens can be divided into reversible deformation (elastic deformation) and irreversible deformation (plastic deformation). In the reversible deformation process, energy is primarily converted into elastic strain energy stored within the specimen. When irreversible deformation occurs, in addition to elastic strain energy, the remaining energy is dissipated mainly in the form of plastic deformation, friction, heat radiation, and damage, referred to as dissipated energy^34^. According to the first law of thermodynamics, assuming no heat exchange between the specimen and the external environment during the uniaxial compression test, the total energy input to the test machine is only converted into elastic strain energy and dissipated energy^35^. That is:

1 $$U = U^{e} + U^{d}$$

where $U$ is the total external input energy (kJ/m^3^), $U^{e}$ is the elastic strain energy (kJ/m^3^), $U^{d}$ is the dissipated energy (kJ/m^3^).

During the uniaxial compression test, the calculation of the total energy change in the specimen is given by:

2 $$U = \int\limits_{0}^{{\varepsilon_{c} }} {\sigma \, d\varepsilon }$$

where: $\sigma$ is the axial stress (MPa), $\varepsilon$ is the strain, $\varepsilon_{c}$ is the maximum strain obtained during the test.

According to Hooke's Law, the calculation of the elastic strain energy can be expressed as:

3 $$U^{e} = \frac{{\sigma_{c}^{2} }}{2E}$$

where: $\sigma_{c}$ is the stress peak (MPa), $E$ is the elastic modulus (GPa).

**Figure 17:**
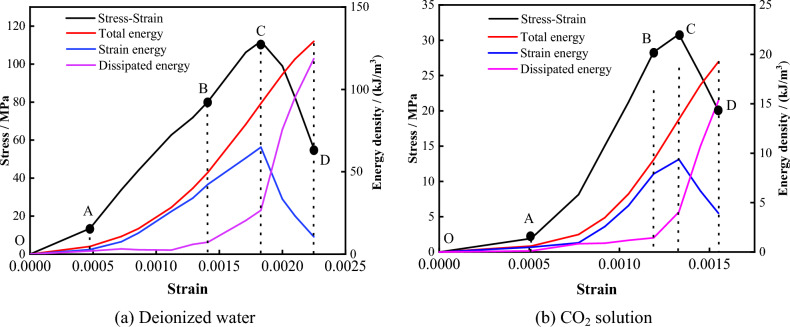
Evolution of stress and energy zoning.

Based on Eqs. (1), (2), and (3), the total energy, elastic strain energy, and dissipated energy of the specimen during uniaxial compression were calculated. By combining the characteristics of the stress–strain curve, the evolution of each energy index with strain was analyzed. Taking the example of 60 days of dissolution, as shown in Fig. 17, the energy evolution process of the limestone specimen can be roughly divided into the following four stages after dynamic dissolution with deionized water or CO_2_ solution:

1. OA Segment: This stage represents the compaction phase of the stress–strain curve. In this stage, a portion of the energy applied by the testing machine is transformed into elastic strain energy, while the other portion is dissipated by the closure of primary fractures. The elastic strain energy is essentially equal to the dissipated energy.
2. AB Segment: With increasing strain, both the total energy and elastic strain energy increase, while the dissipated energy curve remains relatively horizontal. This shows that, in this stage, most of the total energy is converted into elastic strain energy, and the dissipated energy remains essentially constant.
3. BC Segment: As the strain increases, the growth rate of the elastic strain energy decreases, and the growth rate of the dissipated energy increases. The elastic strain energy reaches its maximum value near the peak strength, indicating that the specimen's accumulated energy has reached its limit.
4. CD Segment: With increasing strain, the elastic strain energy sharply decreases, while the dissipated strain energy sharply increases. This suggests that cracks, voids, and other defect areas in the specimen extend and connect after the peak point, causing the rapid release of initially stored elastic strain energy in the form of kinetic energy, thermal energy, etc., ultimately leading to the loss of specimen bearing capacity.

**Figure 18:**
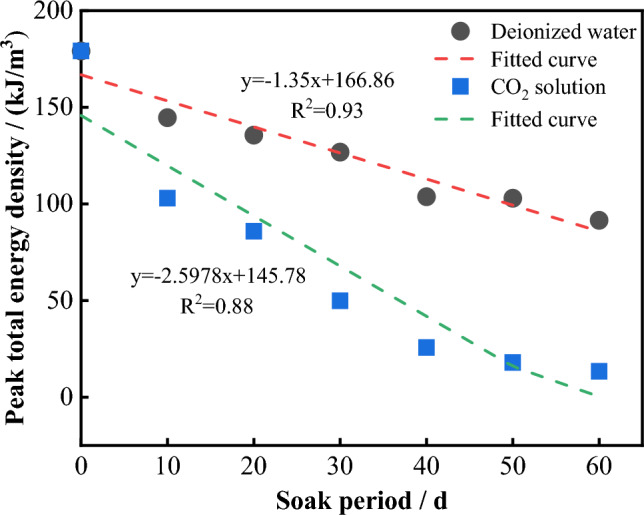
The change of peak total energy with immersion time.

To analyze the variation in specimen energy with immersion time during the erosion process, a relationship between the peak total energy and immersion time was established, as shown in Fig. 18. It can be seen that, with increasing immersion time, the peak total energy of the specimens in both solution environments tends to decrease. The decay rate is more significant in the CO_2_ solution environment, indicating that, throughout the experimental period, the erosion effect of the solution on the specimen remains in a nonequilibrium state. This intensified erosion has deteriorated the rock mass, reducing the energy required for damaging the rock mass absorbed from the outside during the uniaxial compression process. This decrease results in a reduction in the severity of specimen failure, corresponding to the observed characteristics of specimen failure mentioned earlier.

### Parameters of limestone dissolution

During the process of water–rock dissolution, the variations in the dissolution rate and the concentration of major mineral ions can reflect the erosive effects of the solution on the rock mass under different conditions. Based on the mineral analysis mentioned earlier, the main minerals in the limestone in this experiment are calcite (CaCO_3_) and dolomite (CaMg(CO_3_)_2_). Therefore, this study aimed to clarify the material transport patterns during limestone dissolution by monitoring changes in dissolution rates and Ca^2+^ concentrations during different experimental periods.

**Figure 19:**
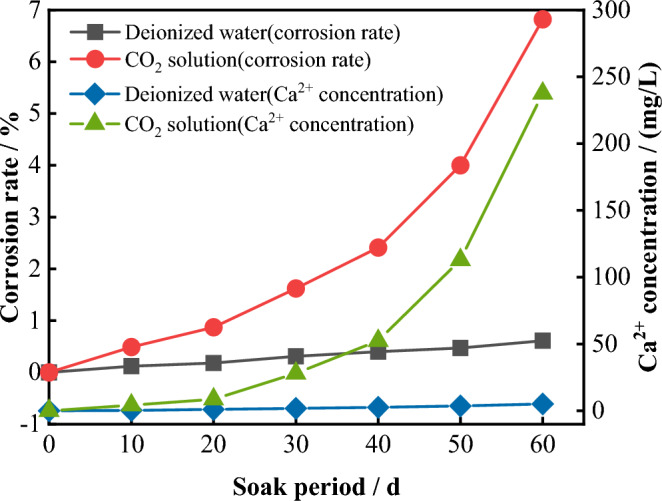
Process of sample dissolution rate and Ca^2+^ concentration changing with immersion time.

The dissolution rate is defined as the ratio of the dissolved mass of the sample after the experiment to the mass of the original sample, expressed by Eq. (4). Figure 19 shows the time evolution curves of the sample dissolution rate and the concentration of Ca^2+^ in the immersed solution. It can be observed that with increasing immersion time, the sample dissolution rate increase. In the deionized water environment, the sample dissolution rate increases slowly with immersion time, reaching a total dissolution rate of 0.61% after 60 days of immersion. In the same experimental period, the increase in the sample dissolution rate after immersion in CO_2_ solution is much greater than that after immersion in deionized water. After 40 days, the sample dissolution rate shows a staged increase, reaching a total dissolution rate of 6.82% after 60 days of immersion, which is 11.18 times greater than that under deionized water conditions. The trend of the Ca^2+^ concentration with that of immersion time is generally consistent with the dissolution rate. In deionized water, the Ca^2+^ concentration also shows a slow increase, with concentrations in each experimental period being 0.30 mg/L, 1.00 mg/L, 1.82 mg/L, 2.61 mg/L, 3.65 mg/L, and 5.15 mg/L, respectively. In CO_2_ solution, the concentrations in each experimental period are 4.18 mg/L, 8.62 mg/L, 28.10 mg/L, 52.41 mg/L, 112.94 mg/L, and 237.61 mg/L. The Ca^2+^ concentrations in each stage are greater than those in the deionized water environment, and the concentration difference gradually increases with increasing immersion time.

4 $$M1 = \frac{{m_{0} - m_{d} }}{{m_{0} }} \times 100\%$$

where: $M1$ is the sample dissolution rate (%), $m_{0}$ is the quality of the undissolved raw sample (g),$m_{d}$ is the quality of the limestone samples d after dissolution (g).

Contrary to previous experimental results reported in the literature, the dissolution rate and Ca^2+^ concentration in this experiment exhibit an opposite trends—they did not gradually decrease during the experimental period. This discrepancy can be attributed to the attempt to simulate the dissolution process of limestone in a real natural environment within a short period. Throughout the experiment, the reaction chamber was kept filled with CO_2_ gas, and the soaking solution was replaced at the end of each experimental stage. Therefore, the dissolution reaction does not slow down gradually due to a reduction in CO_2_ gas or the saturation of the ion concentration in the solution. Instead, it remains in a nonequilibrium state throughout the experiment.

## Degradation mechanism analysis

The erosion of the solution can be divided into physical erosion and chemical erosion. In terms of physics, the lubricating effect of aqueous solution leads to a decrease in the connecting force and friction force between mineral particles. Additionally, the intrusion of aqueous solution brings about pore water pressure, which promotes the development and expansion of internal cracks^36^. From a chemical perspective, certain minerals in rocks react chemically with aqueous solution, thereby altering the size and shape of mineral particles and even destabilizing the stability of mineral structures.

### Chemical corrosion process

Based on the analysis above, the limestone sample is primarily composed of calcite (CaCO_3_) and dolomite (CaMg(CO_3_)_2_), with approximate contents of 80% and 20%, respectively. Both minerals undergo hydrolysis reactions in aqueous solution, and the specific reaction equations are as follows^37,38^:

5 $$\begin{aligned} & CaCO_{3} + H_{2} O \rightleftharpoons Ca^{2 + } + HCO_{3}^{ - } + OH^{ - } \\ & C{\text{a}}M{\text{g}}\left( {CO_{3} } \right)_{2} + 2H_{2} O = C{\text{a}}^{2 + } + M{\text{g}}^{2 + } + 2HCO_{3}^{ - } + 2OH^{ - } \\ \end{aligned}$$

When the experimental chamber is filled with CO_2_ gas, CO_2_ readily dissolves in water to form a carbonic acid (H_2_CO_3_) solution. Subsequently, carbonic acid reacts with calcite and dolomite in the following chemical dissolution reactions:

6 $$\begin{aligned} & H_{2} O + CO_{2} = H_{2} CO_{3} \\ & CaCO_{3} + H_{2} CO_{3} \rightleftharpoons Ca^{2 + } + 2HCO_{3}^{ - } \\ & C{\text{a}}M{\text{g}}\left( {CO_{3} } \right)_{2} + 2H_{2} CO_{3} = C{\text{a}}^{2 + } + M{\text{g}}^{2 + } + 4HCO_{3}^{ - } \\ \end{aligned}$$

**Figure 20:**
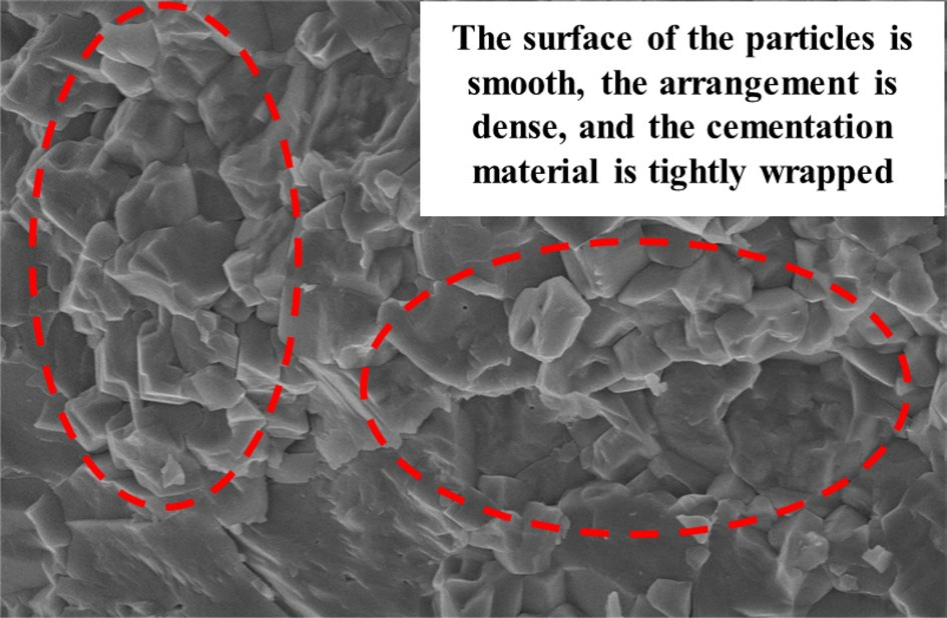
SEM image of undissolved limestone sample.

The fundamental reason for the deterioration in the mechanical properties of the sample due to chemical dissolution is the damage to the microstructure of the sample caused by the chemical reaction between the solution and the rock minerals. Therefore, scanning electron microscopy observations were conducted on the surfaces of the samples without dissolution treatment and under different dynamic dissolution conditions. Figure 20 shows the microstructure morphology of the limestone sample without dissolution treatment. It can be seen that, the microstructure surface of the sample is relatively smooth and flat. The mineral particles are arranged neatly and uniformly, and are tightly enveloped by cementitious material, presenting an overall dense structure.

**Figure 21:**
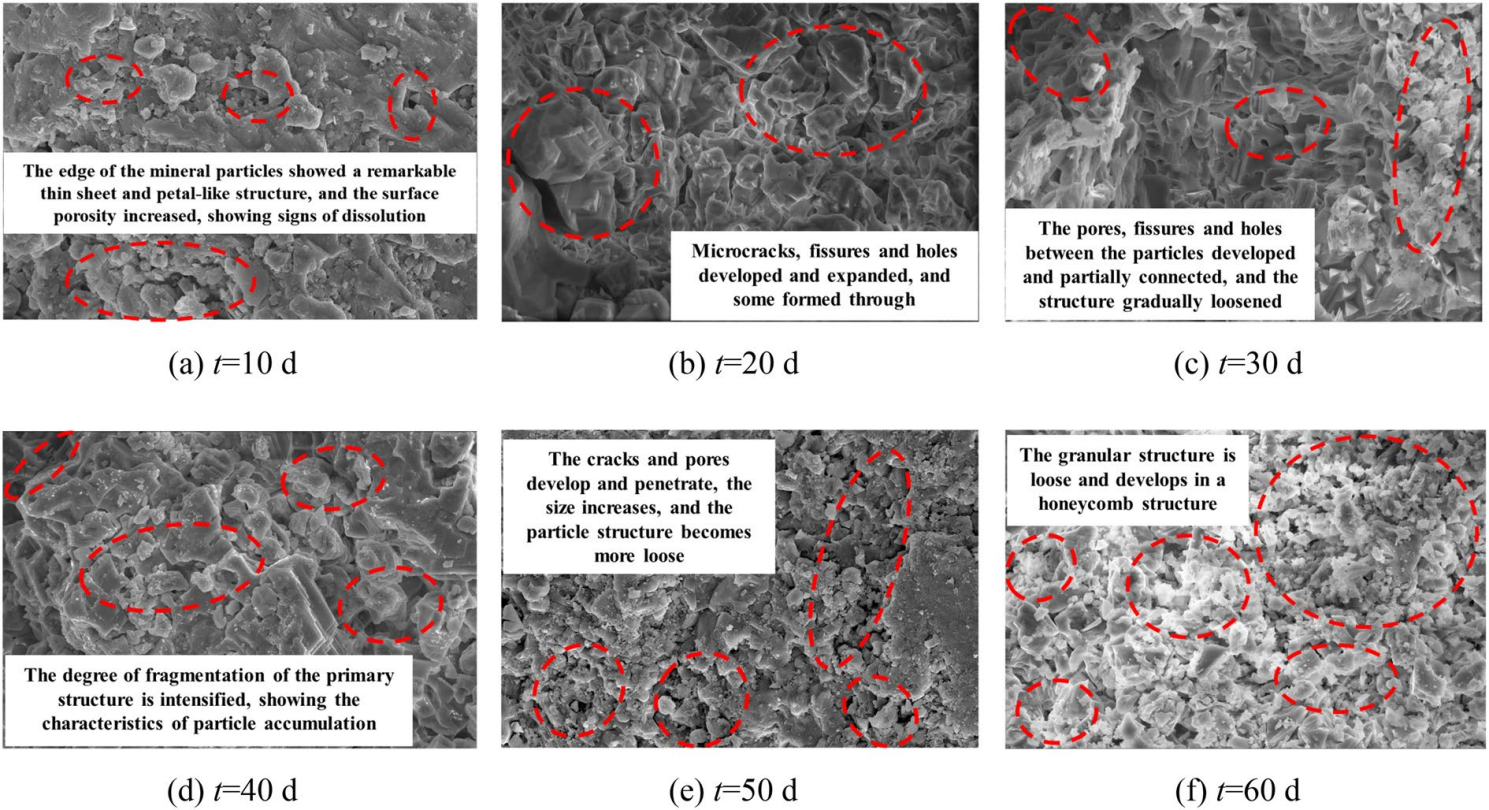
SEM images of limestone samples in CO_2_ solution environment.

As the temporal variation patterns of the microstructure under deionized water and CO_2_ solution environments are roughly similar, and the microstructural changes in the CO_2_ solution environment are more pronounced and easier to observe, so we use the example of the CO_2_ solution environment to illustrate the microstructure images of the samples under different immersion periods, as shown in Fig. 21. It can be seen that, at the beginning of the experiment, the minerals on the sample surface underwent chemical dissolution reactions with the CO_2_ solution, leading to the development of microcracks and dissolution cavities in some areas, providing channels for the gradual expansion of the solution into the interior of the sample. With increasing immersion time, more matrix minerals participated in chemical dissolution reactions, disrupting the connection and arrangement of particles between them. The volume of the expanding defect areas such as pores, cracks, and cavities gradually increased, and some of them formed continuous paths. The surface structure tended to loosen, developing into a porous and loose honeycomb-like structure.

**Figure 22:**
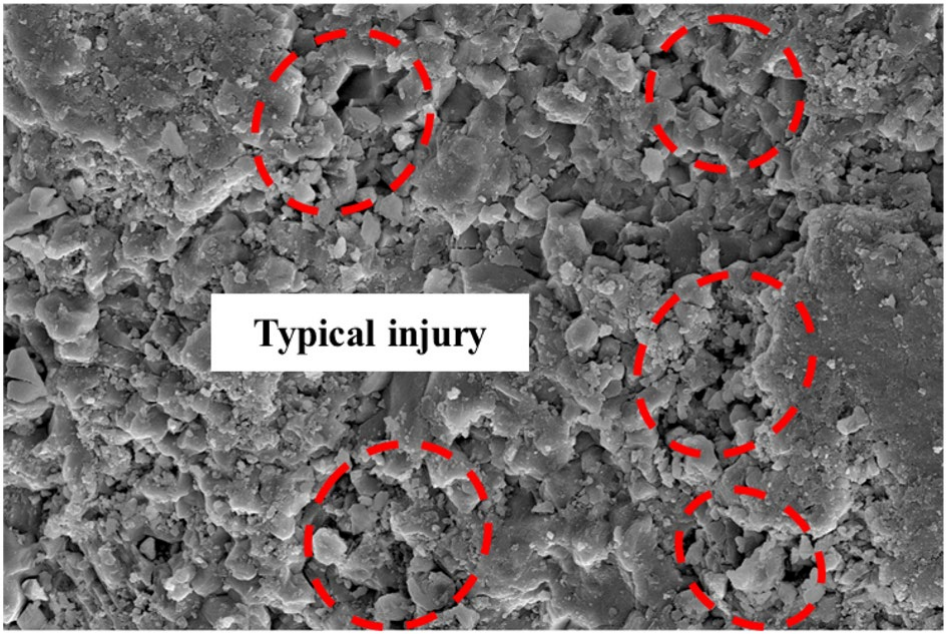
SEM images of typical damage areas.

**Figure 23:**
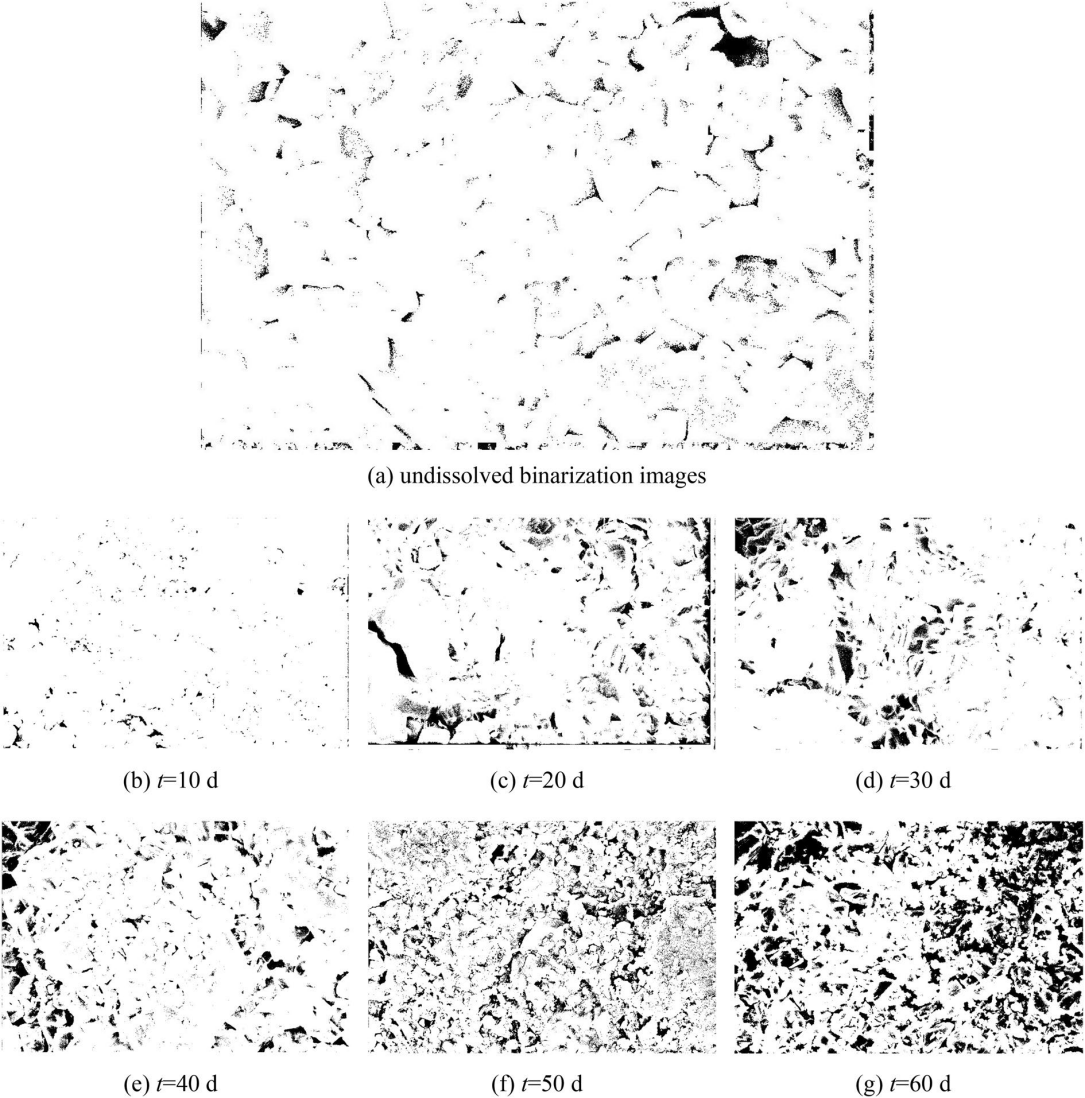
Image of binary processing of limestone samples in CO_2_ solution environment.

To quantitatively evaluate the chemical dissolution effects on the microstructure of the limestone samples from a microscopic perspective, digital image processing techniques were applied to the binary process SEM images. This involved locally enlarging typical damaged areas in the SEM images (as shown in Fig. 22), precisely querying their grayscale information to determine a segmentation threshold. Using this threshold, the content of the SEM images are segmented into two parts: damaged areas such as cracks and cavities formed by chemical reactions and the undamaged rock matrix. Figure 23 shows the SEM image after binary processing, where black represents areas of chemical dissolution damage, such as cracks and cavities, and white represents the rock matrix. Subsequently, based on the binary image, statistical analysis was conducted on the black defect areas, calculating their proportion to quantitatively analyze the impact of chemical dissolution on the damage state of the microstructure of the samples.

**Figure 24:**
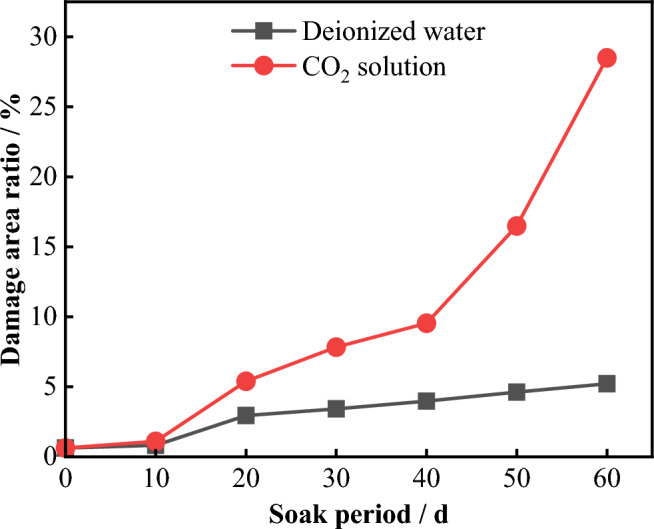
Changes in the damage area ratio over time.

The cracks, pores, and other damaged areas of the samples microstructure were statistically analyzed under different dissolution periods to calculate the ratio of the damaged area to the entire area, namely the damage area ratio, which was used as an indicator to evaluate the deterioration of the damage. Figure 24 shows the temporal evolution curve of the damage area ratio of the sample under different experimental conditions. It can be seen that, the damage area ratio of the samples soaked in deionized water and CO_2_ solution both increased to varying degrees with increasing dissolution period. After 60 days of dissolution, the damage area ratio of the sample soaked in deionized water was 5.22%, while that of the sample soaked in CO_2_ solution was 28.49%.

**Figure 25:**
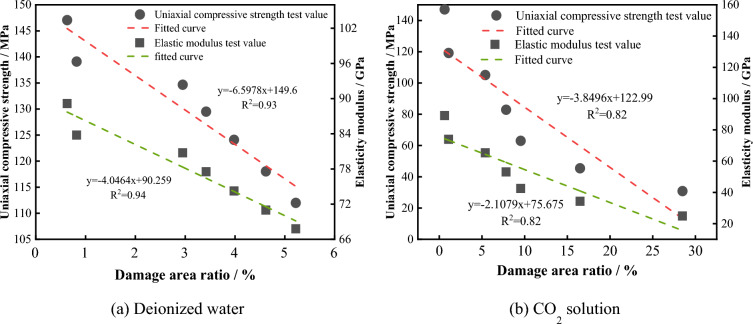
Relation between the damage area ratio and mechanical properties.

**Figure 26:**
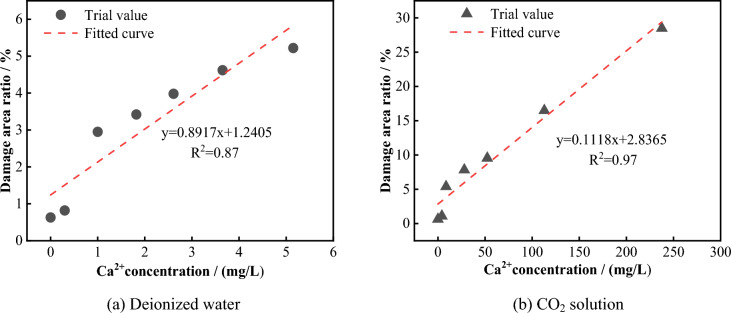
Relationship between Ca^2+^ concentration and damaged area.

To investigate the deterioration mechanism of the mechanical properties of the sample caused by chemical dissolution of the solution and to analyze the correlation between the microstructural characteristics of the solution corrosion and the macroscopic mechanical strength, the relationships between the damage area ratio in the SEM images and the uniaxial compressive strength and elastic modulus were established, as shown in Fig. 25. It can be seen that, the damage area ratio of the samples soaked in deionized water and CO_2_ solution is negatively correlated with the uniaxial compressive strength and elastic modulus, indicating that the deterioration of the mechanical properties of the limestone is controlled by the proportion of the damaged area in the microstructure and that the development of the damaged area is related to the dissolution state of the main rock-forming minerals. Figure 26 shows the linear fitting relationship between the concentration of Ca^2+^ in the solution and the damage area ratio of the samples under two different solution environments. It can be seen that, the damage area ratio is proportional to the concentration of Ca^2+^ released, indicating that the chemical reaction of calcite and dolomite (containing Ca minerals) directly affect the development of the damaged area. Therefore, after long-term soaking in solution, the mineral matrix inside the sample chemically reacts with the solution, and the main mineral elements are precipitated from the structure in the form of ions, causing cracks, fissures, pores, and other defect areas to continuously emerge, expand, and penetrate in the microstructure of the rock, thereby destroying its microstructural stability and ultimately resulting in the deterioration of the macroscopic mechanical properties of the rock mass.

### Solution weakening effect

In the above analysis, the chemical dissolution mechanism of the solution on the limestone samples was described in detail. However, in the water–rock interaction process, both physical and chemical dissolution occur almost simultaneously. The difference lies in the significant variations in the deterioration effects produced by these two kinds of dissolution due to different solution environments. Therefore, to quantify the deterioration effects of physical dissolution and chemical dissolution on sample mechanical properties, an analysis and discussion were conducted from the perspective of changes in dissolution capacity.

Since deionized water was used as the immersion solution in the experiment, the Ca^2+^ in the solution came solely from chemical reactions. Therefore, Ca^2+^ can be used as an indicator to investigate chemical dissolution. By substituting Ca^2+^ into chemical Eqs. (5), (6) and combining with Eq. (7), the chemical dissolution amounts (consumption of CaCO_3_ and CaMg(CO_3_)_2_) under different solution environments were calculated. Correspondingly, the physical dissolution amount under the respective conditions was obtained.

7 $$Ma = M - Mc$$

where: $Ma$ is the amount of physical dissolution (g), $M$ is the total corrosion (g), $Mc$ is the amount of chemical dissolution (g).

**Figure 27:**
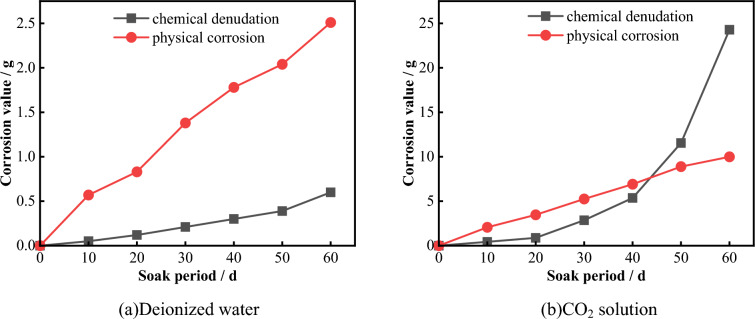
Variation in the physical and chemical dissolution amount of the sample with time.

Figure 27 shows the changes in the chemical and physical erosion amounts of limestone after immersion for different durations in different solution environments. Figure 27a shows that under a deionized water environment, both the chemical and physical erosion amounts of limestone gradually increase with increasing immersion time. However, the physical erosion amount is always greater than the chemical erosion amount at each stage, indicating that under a deionized water environment, physical erosion plays a more dominant role than chemical erosion.

Figure 27b shows that in the early stage of the experiment (0-30d), the physical erosion amount of the limestone sample soaked in CO_2_ solution was slightly greater than the chemical erosion amount. However, after 40 days, the chemical erosion degree increased sharply, and the erosion amount was much greater than the physical erosion amount, this difference rapidly increased with increasing immersion time. During the same experimental period, the physical and chemical erosion amounts of the sample were greater than those in the deionized water environment. After 60 days of immersion, the physical and chemical erosion amounts of the sample were 9.99 mg and 24.28 mg, respectively, which were 3.97 times and 40.36 times greater than those in the deionized water environment.

The above phenomenon indicates that the chemical reaction between the CO_2_ solution and limestone minerals requires a certain reaction time. Due to the chemical erosion of CO_2_ solution, some mineral elements enter the solution and some areas become loose in structure, which makes it easy for them to fall off during the experiment. Therefore, the physical and chemical erosion amounts of the sample are both greater than those in the deionized water environment.

To further analyze the controlling effects of physical and chemical erosion on the deterioration of the sample, the relationships between the Ca^2+^ concentration, amount of erosion, uniaxial compressive strength, and elastic modulus were established based on Eqs. (8), (9), (10), and (11).

8 $$M_{{C{\text{a}}^{2 + } }} = M_{T} - M_{T - 1}$$

where: $M_{{C{\text{a}}^{2 + } }}$ is the Ca^2+^ phase release (mg/L), $M_{T}$ is the Ca^2+^ concentration (mg/L) after* T* test cycles,$M_{T - 1}$ is the Ca^2+^ concentration (mg/L) in the previous test cycle.

9 $$M_{n} = \frac{{Mc_{T} - Mc_{T - 1} }}{{M_{T} - M_{T - 1} }} \times 100\%$$

where: $M_{n}$ is the phase chemical corrosion ratio (%), $M_{T}$ is the total corrosion amount of the sample (g) after T test cycles, $M_{T - 1}$ is the total corrosion amount of the sample (g) in the previous test cycle, $Mc_{T}$ is the chemical corrosion of the sample (g) after *T* test cycles, $Mc_{T - 1}$ is the chemical corrosion of the sample (g) in the previous test cycle.

10 $$M_{\sigma } = \frac{{\sigma_{T - 1} - \sigma_{T} }}{{\sigma_{T - 1} }} \times 100\%$$

where: $M_{\sigma }$ is the uniaxial compressive strength stage degradation rate (%), $\sigma_{T}$ is the uniaxial compressive strengthof the sample (MPa) after *T* test cycles, $\sigma_{T - 1}$ is the uniaxial compressive strength of the sample (MPa) in the previous test cycle.

11 $$M_{E} = \frac{{E_{T - 1} - E_{T} }}{{E_{T - 1} }} \times 100\%$$

where: $M_{E}$ is the elastic modulus stage degradation rate (%), $E_{T}$ is the elastic modulus of the sample (GPa) after *T* test cycles, $E_{T - 1}$ is the elastic modulus of the sample (GPa)in the previous test cycle.

**Figure 28:**
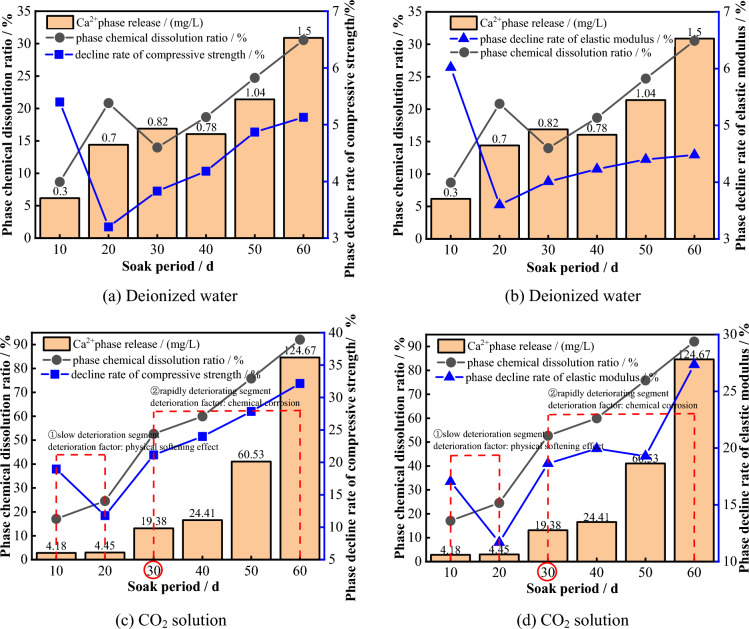
The corresponding relationship between the mechanical properties and the proportion of stage chemical dissolution and Ca^2+^ concentration.

Figure 28 shows the corresponding relationships between the stage chemical erosion ratio, uniaxial compressive strength and elastic modulus deterioration rate, and the concentration change of Ca^2+^ at different soaking times under different solution environments. It can be seen that, in a deionized water environment, the deterioration amplitude of the uniaxial compressive strength and elastic modulus of the sample shows a skewed “V” shaped distribution, and the deterioration amplitude reaches a maximum after 10 days of dissolution, while the stage release amount of Ca^2+^ and the stage chemical erosion ratio are the smallest. In the 20 -60 days stage, the stage deterioration rate of the uniaxial compressive strength and elastic modulus of the sample slowly increases with increasing Ca^2+^ release concentration, but the stage chemical erosion ratio reaches only 30.55%. It can be seen that under deionized water environment, the deterioration of the mechanical properties of the limestone sample is mainly controlled by the physical softening effect of the solution, and this softening effect has obvious “aging”, which basically completes the influence on the macroscopic mechanical properties of the sample in the early stage of the experiment.

For the CO_2_ solution environment, the deterioration process of the mechanical properties of the samples can be divided into two stages:

1. In the 10–20 days stage, the amount of Ca^2+^ released and the proportion of chemical dissolution are relatively small. This suggests that during this stage, the deterioration of the mechanical properties of the limestone sample is mainly controlled by the physical softening effect of the solution.
2. In the 30–60 days stage, with increasing in the release concentration of Ca^2+^, the rate of decrease in the uniaxial compressive strength and elastic modulus becomes significantly larger. The proportions of chemical dissolution in this stage reach 52.66%, 59.97%, 75.82%, and 92.06%, indicating that during this stage, the degradation of the mechanical properties of the limestone sample is mainly controlled by the chemical corrosion of the CO_2_ solution. Moreover, the deterioration effect of chemical dissolution is far greater than that of physical softening.

Therefore, for water–rock reactions controlled by the CO_2_ reaction mechanism, the degradation of mechanical properties does not solely depend on the chemical dissolution of the main minerals. At the early stage of the experiment, the degree of chemical dissolution reaction is low and the deterioration of the macro-mechanical properties of the sample mainly depends on the mechanical dissolution and physical softening of the aqueous solution because it takes time for chemical reactions between the CO_2_ solution and limestone minerals to occur. As time goes by, the degree of chemical reaction intensifies, resulting in the release of a large amount of Ca^2+^ into the solution. This causes the disintegration of mineral structures and ultimately results in a significant deterioration of the macroscopic mechanical properties of the sample. Therefore, the degradation of the macroscopic mechanical properties primarily depends on the chemical dissolution caused by the CO_2_ solution at the later stages of the experiment. Furthermore, the degradation rate caused by chemical dissolution is significantly greater than that caused by physical softening in the earlier stages.

## Conclusion

In this paper, limestone is taken as the research object, and dynamic dissolution experiments are carried out in deionized water and CO_2_ solution, respectively, to explore the influence of solution properties and soaking time on the mechanical properties of limestone. Based on the changes in microstructure, erosion rate, and Ca^2+^ concentration, the deterioration mechanism of limestone under different reaction processes is analyzed. The main conclusions are as follows:

1. The uniaxial compressive strength and elastic modulus of the samples in both solution environments decrease with increasing immersion time. However, the deterioration degree of the samples in CO_2_ solution is greater than that in deionized water, and this difference gradually increases with increasing of immersion time.
2. During the uniaxial compression test, the failure mode of the sample was mainly brittle tensile failure, and its ductility property gradually increased with increasing immersion time. Based on the energy method and the characteristics of the stress–strain curve, the evolution of the elastic strain energy and dissipation energy of the sample during the compression process can be divided into four stages. The total energy of the peak stress decreases gradually with increasing immersion time, and the attenuation rate in the CO_2_ solution is greater than that in the deionized water, indicating that the dynamic dissolution effect of the solution causes deterioration inside the rock, which in turn leads to a decrease in the energy required for rock mass failure.
3. In deionized water, the dissolution of limestone occurs mainly through ionization hydrolysis of calcite and dolomite, while in CO_2_ solution, the chemical reaction between calcite, dolomite and H^+^ is mainly involved. During the dissolution process, the dissolution rate and Ca^2+^ concentration of the sample increase with increasing immersion time. However, during the same experimental period, the stage growth rate of the dissolution rate and Ca^2+^ concentration after soaking in CO_2_ solution is much greater than that in the deionized water environment.
4. In both types of solution environments, the damage area of the sample increases with increasing immersion time, and is positively correlated with the Ca^2+^ concentration. It is negatively correlated with the uniaxial compressive strength and elastic modulus. This indicates that the chemical deterioration effect of the solution on the sample is mainly controlled by the release degree of Ca^2+^ in the mineral structure.
5. In deionized water, the deterioration of the mechanical properties of limestone is mainly controlled by mechanical erosion and physical softening. In CO_2_ solution, the deterioration of the mechanical properties of limestone can be divided into two stages. In the early stage of the experiment, the deterioration of limestone is also controlled by physical erosion. However, with the passage of the experimental period, chemical erosion gradually becomes the main controlling factor, and its deterioration effect is much greater than that of physical deterioration.

## Data Availability

The datasets generated during and analysed during the current study are available from the corresponding author on reasonable request.
